# The impact of intrauterine adhesions on endometrial receptivity in patients undergoing *in vitro* fertilization-embryo transfer

**DOI:** 10.3389/fendo.2024.1489839

**Published:** 2025-01-21

**Authors:** Yan Ouyang, Yangqin Peng, Mingxiang Zheng, Yuyao Mao, Fei Gong, Yuan Li, Hui Chen, Xihong Li

**Affiliations:** ^1^ Clinical Research Center for Reproduction and Genetics in Hunan Province, Reproductive & Genetic Hospital of CITIC-Xiangya, Changsha, China; ^2^ College of Information Science and Engineering, Hunan University, Changsha, China; ^3^ NHC Key Laboratory of Human Stem Cell and Reproductive Engineering, School of Basic Medical Sciences, Central South University, Hunan University, Changsha, China

**Keywords:** endometrial receptivity, intrauterine adhesion, frozen-thawed embryo transfer, natural cycle, ovulation day, transvaginal sonography

## Abstract

**Objective:**

To clarify whether intrauterine adhesions (IUAs) affect endometrial receptivity (ER) on the day of ovulation and to compare patients with mild and moderate-severe adhesions.

**Methods:**

This prospective cohort study included 592 infertile women with IUAs who underwent frozen-thawed embryo transfer (FET). Patients were divided into groups with or without IUAs; and pregnant and nonpregnant populations based on whether a clinical pregnancy was achieved. The ultrasound ER parameters on the ovulation day were compared. Patients with IUAs were then divided into mild or moderate-severe IUA subgroups according to IUA degree.

**Results:**

The proportions of patients with Type B plus Type C endometrial morphology (94% vs. 75%, P<0.001), an endometrial thickness≥8mm (97% vs. 81%, P<0.001), an endometrial volume≥2ml (94% vs. 67%, P<0.001), a frequency of endometrial peristalsis≥2 times/min (84% vs. 53%, P<0.001), low subendometrial volume (11.54 ± 2.94 vs. 9.57 ± 2.35, P<0.001) and subendometrial vascularization flow index (VFI) values (2.70 ± 3.10 vs. 2.23 ± 2.23, P=0.033) and a low live birth rate (65% vs. 56%, P=0.039) were significantly higher in the group without IUAs than in the group with IUAs. The group with moderate-severe IUAs had lower proportion of patients with an endometrial thickness≥8mm (73% vs. 89%, P=0.008) and an endometrial volume ≥2ml (58% vs. 78%, P=0.005), a lower frequency of endometrial peristalsis≥2 times/min (42% vs. 65%, P=0.003), and low subendometrial volume (9.22 ± 2.29 vs. 9.97 ± 2.36, P=0.023) and subendometrial flow index (FI) (31.48 ± 3.64 vs. 33.43 ± 4.17, P=0.002) values than the group with mild IUAs; a high antral follicle count (AFC), basal follicle-stimulating hormone (FSH), and anti-Müllerian hormone (AMH) levels and an endometrial thickness≥8mm were independent predictors of clinical pregnancy.

**Conclusion:**

IUAs can affect ER on the ovulation day and the live birth rate during natural cycles. Moderate-severe IUAs have a greater impact on ER than mild adhesions do; however, if these adhesions are treated properly, they do not have adverse effects on the clinical pregnancy rate. A high AFC, basal FSH and AMH levels and an endometrial thickness ≥8 mm were found to be independent predictors of clinical pregnancy.

## Introduction

Intrauterine adhesions (IUAs) are considered one of the main reproductive system diseases in women worldwide and are characterized by endometrial fibrosis with partial to complete obliteration of the uterine cavity and/or cervical canal (1, 2). The reported prevalence of IUAs varies between 0.3% and 21.5% (3). Any event that causes damage to the endometrium may lead to the development of IUAs, resulting in menstrual disturbances, infertility and (recurrent) pregnancy loss (4).

Transvaginal sonography (TVS) has been adopted as a routine method to assess endometrial receptivity (ER) (5). Hysteroscopic adhesiolysis is the standard treatment for removing IUAs, restoring the uterine architecture and facilitating communication among the uterine cavity, cervical canal and fallopian tubes to allow both normal menstrual flow and adequate sperm transportation (6). While B Pecorino et al. (7) reported that endometrial scratching in the patients with repeated implantation failure had no significant improvement in implantation and clinical pregnancy rates.

The relationship between IUAs and reproductive performance has been frequently described in the literature; IUAs, especially moderate and severe IUAs, may strongly impact fertility, predisposing individuals to pregnancy disorders and obstetric complications in subsequent pregnancies (8–10). Is this because IUAs have a negative impact on ER? The aim of the present study was to clarify whether IUAs affect ER on the day of ovulation and to further compare patients with mild and moderate–severe adhesions. To our knowledge, this is the first prospective study comparing ER on the day of ovulation during natural cycles between patients with and without a history of adhesions.

## Materials and methods

### Study design and participants

This prospective cohort study was conducted at the Reproductive and Genetic Hospital of CITIC-Xiangya from March 2019 to September 2023, and 592 infertile women (417 with a history of IUAs and 175 without no IUAs) who underwent frozen-thawed embryo transfer (FET) were included. Written informed consent was obtained from all participants. The institutional review board approved this study (date of approval: 11 September 2019; reference number: LL-SC-2019-023; Changsha, China).

The inclusion criteria for patients with a history of IUAs were as follows (1): underwent FET after adhesiolysis (2); underwent a natural FET cycle (3); aged 20–35 years (4); had a body mass index of 18–24 kg/m^2^; and (5) had at least 1 high-quality embryo. The exclusion criteria were as follows (1): endometriosis, adenomyosis, or adenomyoma (2); endometritis (3); congenital uterine malformations (4); untreated hydrosalpinx; or (5) uterine cavity fluid (diameter ≥2 mm) caused by caesarean section diverticulum.

The inclusion criteria for the control group of patients with no IUAs were as follows (1): underwent FET (2); underwent a natural FET cycle (3); age 20-35 years (4); body mass index 18-24 kg/m2 (5) had at least 1 high-quality embryo. The exclusion criteria were (1): endometriosis, adenomyosis, or adenomyoma (2); intrauterine adhesion or endometritis (3);congenital uterine malformations (4); untreated hydrosalpinx; or (5) uterine cavity fluid caused by cesarean section diverticulum and diameter ≥2 mm.

### Adhesiolysis

IUAs were graded on the basis of the American Fertility Society classification (11): mild IUA was indicated by 1-4, moderate was indicated by 5-8, and severe was indicated by 9-12. All the included patients underwent adhesiolysis in our center before the *in vitro* fertilization (IVF) procedure, which was performed by an experienced surgeon under general anesthesia. The adhesions were dissected by using bipolar energy (Olympus) and/or hysteroscopic scissors (Karl Storz, Tuttlingen, Germany) until the uterine cavity was achieved to restore uterine morphology. Then, based on patients’ uterine width, a heart-shaped intrauterine balloon (Cook Medical, Bloomington, IN, USA) or Foley catheter was inserted into the uterine cavity, and all patients received crosslinked hyaluronan gel (MateRegen; BioRegen Biomedical, Changzhou, China) to prevent adhesion reformation.

### *In vitro* fertilization procedure

Depending on the cause of infertility, IVF or intracytoplasmic sperm injection (ICSI) was applied for fertilization. In this study, all patients underwent FET during natural cycles. Thawed cleavage-stage embryos and blastocysts were transferred on the 3rd day and the 5th day after ovulation. Embryo morphology was scored on the basis of the Alpha Scientists in Reproductive Medicine and European Society of Human Reproduction and Embryology (ESHRE) Special Interest Group of Embryology (ASEBIR) consensus (12). A maximum of two embryos were transferred.

### Ultrasound evaluation

#### Ultrasound diagnosis of IUAs

GE VOLUSON E8 ultrasound instrument (GE VOLUSON, E8, General Electric Tech Co., Ltd., New York, USA) equipped with a 5–9 MHz transvaginal three-dimensional (3D) probe was used for the preoperative evaluation of uterine cavity. 2D ultrasound was used routinely as a first-line diagnostic tool for the assessment of the endometrial integrity to look for disruptions of the endometrial–myometrial junction. Adhesions are seen as bands of myometrial tissue traversing the endometrial cavity and adjoining the opposing uterine walls. For suspected patients, 3D ultrasound was used to further clarify adhesions. During 3D TVS, the morphological characteristics suggesting IUA were classified into six categories: marginal irregularity, thinning (< 2 mm), defect, obliteration, fibrosis, or calcification (13, 14).

### Assessment of ER

TVS was performed on the day of ovulation for all included patients to evaluate ER. Ultrasound ER parameters, including endometrial thickness, morphology, volume, movement and blood perfusion, were assessed by the same senior ultrasonic doctor (Dr. Li) using the same ultrasound machine.

The maximum diameter of the endometrium was measured in the longitudinal plane. The Gonen classification criteria (15) were adopted to classify endometrial morphology: Type A: the increase in reflectivity leads to a completely homogeneous, hyperechoic endometrium, with the central echo line not visible; Type B: the endometrium has the same reflectivity compared to the surrounding myometrium, and the central echo line is not obvious or missing; and Type C: a “triple-line” endometrium is present, consisting of a prominent outer and central hyperechogenic line and inner hypoechogenic or black regions.

The movement of the endometrium was observed and recorded within 3 minutes and was divided into 5 types according to Ljland et al. (16) (1): positive wave: the peristaltic wave from the cervix to fundus (2); negative wave: the peristaltic wave from the fundus to the cervix (3); static wave: the endometrium is in a static state (4); bidirectional wave: the endometrium of both the uterine fundus and cervix contract simultaneously; and (5) random wave: irregular motion types with an uncertain direction or multiple starting points.

Endometrial blood perfusion was evaluated based on the Applebaum classification standard (17): I: vessels penetrate the outer hypoechoic area around the endometrium but do not enter the outer edge of the hyperechoic area; II: vessels penetrate the outer edge of the endometrium with high echogenicity but do not enter the internal area of low echogenicity; and III: vessels enter the hypoechogenic inner area of the endometrium.

The ultrasound machine was switched to 3D mode with power Doppler. Virtual organ computer-aided analysis (VOCAL) software was used to outline the endometrium, and the endometrial volume, endometrial vascularization index (VI), vascularization flow index (VFI) and flow index (FI) were obtained (18, 19).

### Outcome measures

Serum human chorionic gonadotropin (HCG) levels were measured 14 days (12 days after blastocyst transfer), and TVS was performed 4 weeks after transfer. The primary outcome was live birth, defined as the complete expulsion or extraction of a product of fertilization after 20 completed weeks of gestation that, after separation from the woman, breathed or showed other evidence of life, irrespective of whether the umbilical cord had been cut or the placenta was attached. The secondary outcome was clinical pregnancy, which was confirmed if a gestational sac was observed, and a viable pregnancy was diagnosed when fetal cardiac activity was detected (20).

The included patients were divided into groups according to the presence or absence of IUAs: the group with IUAs and the group without IUAs. All enrolled patients were further divided into two groups according to whether a clinical pregnancy was achieved: the pregnant group and the nonpregnant group. Patients with IUAs were then divided into mild or moderate-severe IUA subgroups according to the degree of IUAs. The ER parameters on the day of ovulation were compared between these groups.

### Statistical analysis

The distribution of patient demographics was analyzed via the Kolmogorov–Smirnov test. Continuous variables are expressed as means ± standard deviations (SDs). Categorical variables are described as frequencies and percentages. The Mann−Whitney U test or Student’s t test was used to assess continuous variables, and the chi-square test or Fisher’s exact test was used to assess differences in categorical variables between the pregnant group and the nonpregnant group. Univariate and multivariate logistic regression analyses were used to identify independent predictors of clinical pregnancy; odds ratios (ORs) and 95% confidence intervals (CIs) were calculated. All the statistical analyses were performed via R software (version 4.3.2), and a two-sided p value < 0.05 was considered statistically significant.

## Results

### Characteristics and ultrasound parameters on the day of ovulation for patients without IUAs

During the study period, there were a total of 191 patients with a history of IUAs, after excluding 10 patients who were dropped and 6 patients of lost to follow up, 175 patients with previous IUAs (93 patients had moderate–severe IUAs, and 82 patients had mild IUAs) were included; A total of 447 patients who did not have a history of IUAs were enrolled, after excluding 19 patients who were dropped and 11 patients of lost to follow up, 417 patients were ultimately included as the control group. The overall clinical pregnancy rates were 69.9% (414/592) and 72.2% (301/417) for patients without IUAs, 64.6% (113/175) for patients with a history of IUAs [63.4% (59/93) for moderate-severe IUAs, 65.9% (54/82) for mild IUAs]. The basic, clinical and endometrial ultrasound characteristics of the overall group, pregnant group and nonpregnant group of patients without or with IUAs are displayed in **Table 1**.

**Table 1:** Comparisons of characteristics and ultrasound parameters on the day of ovulation.

Variable	Non-IUAs			IUAs			p value				
	1	2	3	4	5	6	1 vs. 4	2 vs. 3	5 vs. 6	2 vs. 5	3 vs. 6
	Overall	Nonpregnant group	Pregnant group	Overall	Nonpregnant group	Pregnant group					
Cycles	417	116	301	175	62	113					
Baseline characteristics											
Age (years)	30.22 ± 3.17	30.19 ± 3.34	30.23 ± 3.12	30.34 ± 2.84	30.56 ± 2.72	30.22 ± 2.90	0.911	0.814	0.447	0.641	0.786
Duration of infertility (years)	3.59 ± 2.35	3.93 ± 2.91	3.47 ± 2.11	2.91 ± 1.88	2.84 ± 1.88	2.94 ± 1.89	<0.001	0.368	0.693	0.012	0.017
BMI (kg/m2)	21.20 ± 1.71	21.00 ± 1.74	21.28 ± 1.69	20.94 ± 1.67	20.99 ± 1.49	20.91 ± 1.77	0.067	0.082	0.605	0.903	0.034
Number of oocyte retrieval cycles	1.14 ± 0.37	1.21 ± 0.43	1.11 ± 0.35	1.17 ± 0.42	1.18 ± 0.43	1.16 ± 0.41	0.555	0.011	0.737	0.535	0.301
AFC	18.18 ± 14.57	15.03 ± 11.85	19.32 ± 15.29	19.57 ± 14.38	15.60 ± 17.07	21.74 ± 12.21	0.190	0.024	<0.001	0.476	0.022
FSH	5.62 ± 2.31	6.19 ± 2.94	5.42 ± 2.01	5.88 ± 2.03	6.08 ± 2.00	5.77 ± 2.05	0.098	0.001	0.327	0.935	0.075
LH	5.34 ± 10.96	6.25 ± 11.54	5.01 ± 10.75	5.05 ± 6.02	5.53 ± 7.26	4.79 ± 5.24	0.014	0.107	0.782	0.401	0.023
AMH	4.92 ± 3.78	4.88 ± 3.39	4.94 ± 3.92	5.14 ± 3.63	4.91 ± 3.97	5.26 ± 3.44	0.354	0.701	0.152	0.534	0.102
Ultrasound indicators on the day of ovulation											
Endometrial morphology classification							<0.001	0.66	0.034	<0.001	<0.001
Type A	25 (6.0%)	6 (5.2%)	19 (6.3%)	43 (25%)	21 (34%)	22 (19%)					
Type B+Type C	392 (94%)	110 (95%)	282 (94%)	132 (75%)	41 (66%)	91 (81%)					
Endometrial blood flow classification							0.070	0.780	0.197	0.026	0.702
I	37 (8.9%)	10 (8.6%)	27 (9.0%)	23 (13%)	11 (18%)	12 (11%)					
II	330 (79%)	90 (78%)	240 (80%)	140 (80%)	49 (79%)	91 (81%)					
III	50 (12%)	16 (14%)	34 (11%)	12 (6.9%)	2 (3.2%)	10 (8.8%)					
Endometrial thickness (mm)	11.21 ± 2.08	11.29 ± 2.23	11.18 ± 2.02	9.53 ± 1.93	9.57 ± 2.41	9.50 ± 1.62	<0.001	0.613	0.767	<0.001	<0.001
<8	12 (2.9%)	4 (3.4%)	8 (2.7%)	34 (19%)	20 (32%)	14 (12%)					
≥8	405 (97%)	112 (97%)	293 (97%)	141 (81%)	42 (68%)	99 (88%)	<0.001	0.745	0.001	<0.001	<0.001
Endometrial volume (ml)	4.21 ± 1.72	4.11 ± 1.81	4.24 ± 1.69	2.89 ± 1.45	2.63 ± 1.41	3.03 ± 1.46	<0.001	0.415	0.049	<0.001	<0.001
<2	26 (6.2%)	9 (7.8%)	17 (5.6%)	57 (33%)	28 (45%)	29 (26%)					
≥2	391 (94%)	107 (92%)	284 (94%)	118 (67%)	34 (55%)	84 (74%)	<0.001	0.424	0.008	<0.001	<0.001
Frequency of endometrial peristalsis (time/min)	2.85 ± 1.39	2.87 ± 1.48	2.84 ± 1.36	1.79 ± 1.42	1.57 ± 1.34	1.91 ± 1.46	<0.001	0.767	0.127	<0.001	<0.001
<2	67 (16%)	21 (18%)	46 (15%)	83 (47%)	35 (56%)	48 (42%)					
≥2	349 (84%)	95 (82%)	254 (85%)	92 (53%)	27 (44%)	65 (58%)	<0.001	0.491	0.077	<0.001	<0.001
Endometrial VI	2.13 ± 2.96	2.19 ± 2.67	2.10 ± 3.06	1.95 ± 2.79	1.85 ± 2.28	2.00 ± 3.05	0.228	0.458	0.349	0.126	0.696
Endometrial FI	28.04 ± 6.71	28.86 ± 6.33	27.72 ± 6.83	28.83 ± 6.10	27.92 ± 6.63	29.33 ± 5.75	0.509	0.426	0.041	0.224	0.078
Endometrial VFI	0.81 ± 2.10	0.79 ± 1.19	0.81 ± 2.37	0.69 ± 1.19	0.69 ± 1.31	0.68 ± 1.12	0.460	0.296	0.251	0.122	0.855
Subendometrial volume (ml)	11.54 ± 2.94	11.30 ± 2.82	11.62 ± 2.99	9.57 ± 2.35	9.06 ± 2.36	9.86 ± 2.30	<0.001	0.205	0.046	<0.001	<0.001
Subendometrial VI	7.47 ± 6.60	7.47 ± 5.79	7.48 ± 6.90	6.74 ± 6.46	5.32 ± 5.22	7.52 ± 6.95	0.056	0.55	0.031	0.005	0.759
Subendometrial FI	32.36 ± 4.76	32.84 ± 5.10	32.17 ± 4.61	32.39 ± 4.01	31.62 ± 4.17	32.81 ± 3.87	0.937	0.282	0.053	0.103	0.176
Subendometrial VFI	2.70 ± 3.10	2.57 ± 2.06	2.75 ± 3.42	2.23 ± 2.23	1.71 ± 1.72	2.52 ± 2.42	0.033	0.483	0.023	0.002	0.688
Clinical pregnancy							0.065				
No	116 (28%)			62 (35%)							
Yes	301 (72%)			113 (65%)							
Live birth							0.039				
No	146 (35%)			77 (44%)							
Yes	271 (65%)			98 (56%)							

Patients in the nonpregnant group had greater numbers of oocytes retrieved (1.21 ± 0.43 vs. 1.11 ± 0.35, P=0.011), higher basal follicle-stimulating hormone (FSH, 6.19 ± 2.94 vs. 5.42 ± 2.01, P=0.001) levels and lower antral follicle counts (AFCs, 15.03 ± 11.85 vs. 19.32 ± 15.29, P=0.024) than did those in the pregnant group. There were no significant differences in any endometrial or subendometrial ultrasound indicators on the day of ovulation between the nonpregnant group and the pregnant group.

### Characteristics and ultrasound parameters on the day of ovulation for patients with IUAs

For patients with IUAs, the nonpregnant group had a lower AFC (15.60 ± 17.07 vs. 21.74 ± 12.21, P<0.001), lower proportions of patients with Type B plus Type C endometrial morphology (66% (41/62) vs. 81% (91/113), P=0.034), an endometrial thickness ≥8 mm (68% (42/62) vs. 88% (99/113), P=0.001) and an endometrial volume ≥2 ml (55% (34/62) vs. 74% (84/113), P=0.008), and lower endometrial FI (27.92 ± 6.63 vs. 29.33 ± 5.75, P=0.041), subendometrial volume (9.06 ± 2.36 vs. 9.86 ± 2.30, P=0.046), subendometrial VI (5.32 ± 5.22 vs. 7.52 ± 6.95, P=0.031), and subendometrial VFI (1.71 ± 1.72 vs. 2.52 ± 2.42, P=0.023) values than those in the pregnant group. No significant differences in other ultrasound indicators were found.

### Characteristics and ultrasound parameters on the day of ovulation between patients with and without IUAs

Patients without IUAs generally had a longer duration of infertility (3.59 ± 2.35 years vs. 2.91 ± 1.88 years, P<0.001) and higher basal luteinizing hormone (LH) levels (5.34 ± 10.96 vs. 5.05 ± 6.02, P=0.014) than those with IUAs did; other basic characteristics, such as female age, BMI, number of oocyte retrieval cycles, AFC, FSH level, and anti-Müllerian hormone (AMH) level, were not significantly different between the two groups (P>0.05). In the comparison of the ER ultrasound parameters between the two groups, the proportions of patients with Type B plus Type C endometrial morphology (94% (392/417) vs. 75% (132/175), P<0.001), an endometrial thickness ≥8 mm (97% (405/417) vs. 81% (141/175), P<0.001), an endometrial volume ≥2 ml (94% (391/417) vs. 67% (118/175), P<0.001), a frequency of endometrial peristalsis ≥2 times/min (84% (349/417) vs. 53% (92/175), P<0.001), low subendometrial volume (11.54 ± 2.94 vs. 9.57 ± 2.35, P<0.001) and subendometrial VFI (2.70 ± 3.10 vs. 2.23 ± 2.23, P=0.033) values on the day of ovulation and a low live birth rate (65% (271/417) vs. 56% (98/175), P=0.039) were significantly higher in the group without IUAs than that in the group with IUAs.

For nonpregnant patients, the group without IUAs had a longer duration of infertility, a greater proportion of patients with Type B plus Type C endometrial morphology, greater proportions of patients with an endometrial thickness ≥8 mm, an endometrial volume ≥2 ml, a frequency of endometrial peristalsis ≥2 times/min, a subendometrial volume, a greater proportion of patients with type III endometrial blood flow, and greater subendometrial VFI and VI values than did the group with IUAs.

Compared with patients with IUAs, patients without IUAs also had a longer duration of infertility, a greater BMI, a greater basal LH level, a greater likelihood of having Type B plus Type C endometrial morphology, an endometrial thickness ≥8 mm, and an endometrial volume ≥2 ml, a greater frequency of endometrial peristalsis ≥2 times/min, a greater subendometrial volume, and a lower AFC.

### Characteristics and ultrasound parameters on the day of ovulation between patients with moderate-severe IUAs and patients with mild IUAs

There were no significant differences in any basic characteristics between patients with moderate-severe IUAs and patients with mild IUAs, but patients with moderate-severe IUAs had a lower likelihood of having an endometrial thickness ≥8 mm (73% (68/93) vs. 89% (73/82), P=0.008) and an endometrial volume ≥2 ml (58% (54/93) vs. 78% (64/82), P=0.005), a lower frequency of endometrial peristalsis ≥2 times/min (42% (39/93) vs. 65% (53/82), P=0.003), and lower subendometrial volume (9.22 ± 2.29 vs. 9.97 ± 2.36, P=0.023) and a subendometrial FI (31.48 ± 3.64 vs. 33.43 ± 4.17, P=0.002) values than did patients with mild IUAs (**Table 2**).

**Table 2:** Comparisons of characteristics and ultrasound parameters on the day of ovulation between the mild- and moderate-severe IUA groups.

Variable	Overall	Moderate-to-severe IUAs	Mild IUAs	p value
Cycles	175	93	82	
Baseline characteristics				
Age (years)	30.34 ± 2.84	30.24 ± 2.89	30.47 ± 2.79	0.743
Duration of infertility (years)	2.91 ± 1.88	2.81 ± 1.72	3.01 ± 2.05	0.739
BMI (kg/m2)	20.94 ± 1.67	20.72 ± 1.52	21.20 ± 1.80	0.07
Number of oocyte retrieval cycles	1.17 ± 0.42	1.18 ± 0.44	1.15 ± 0.39	0.606
AFC	19.57 ± 14.38	17.62 ± 13.86	21.77 ± 14.73	0.057
FSH	5.88 ± 2.03	5.80 ± 2.13	5.98 ± 1.93	0.266
LH	5.05 ± 6.02	5.40 ± 7.42	4.66 ± 3.87	0.518
AMH	5.14 ± 3.63	4.82 ± 3.16	5.49 ± 4.08	0.446
Ultrasound indicators on the day of ovulation				
Endometrial morphology classification				0.958
Type A	43 (25%)	23 (25%)	20 (24%)	
Type B+Type C	132 (75%)	70 (75%)	62 (76%)	
Endometrial blood flow classification				0.864
I	23 (13%)	13 (14%)	10 (12%)	
II	140 (80%)	73 (78%)	67 (82%)	
III	12 (6.9%)	7 (7.5%)	5 (6.1%)	
Endometrial thickness (mm)	9.53 ± 1.93	9.07 ± 1.69	10.05 ± 2.07	<0.001
<8	34 (19%)	25 (27%)	9 (11%)	0.008
≥8	141 (81%)	68 (73%)	73 (89%)	
Endometrial volume (ml)	2.89 ± 1.45	2.59 ± 1.29	3.23 ± 1.56	0.002
<2	57 (33%)	39 (42%)	18 (22%)	0.005
≥2	118 (67%)	54 (58%)	64 (78%)	
Frequency of endometrial peristalsis (time/min)	1.79 ± 1.42	1.43 ± 1.26	2.20 ± 1.49	<0.001
<2	83 (47%)	54 (58%)	29 (35%)	0.003
≥2	92 (53%)	39 (42%)	53 (65%)	
Endometrial VI	1.95 ± 2.79	2.13 ± 3.14	1.73 ± 2.34	0.311
Endometrial FI	28.83 ± 6.10	28.15 ± 6.37	29.60 ± 5.72	0.367
Endometrial VFI	0.69 ± 1.19	0.72 ± 1.29	0.65 ± 1.06	0.537
Subendometrial volume (ml)	9.57 ± 2.35	9.22 ± 2.29	9.97 ± 2.36	0.023
Subendometrial VI	6.74 ± 6.46	6.69 ± 6.19	6.79 ± 6.79	0.98
Subendometrial FI	32.39 ± 4.01	31.48 ± 3.64	33.43 ± 4.17	0.002
Subendometrial VFI	2.23 ± 2.23	2.14 ± 2.02	2.33 ± 2.45	0.842
Clinical pregnancy				0.739
No	62 (35%)	34 (37%)	28 (34%)	
Yes	113 (65%)	59 (63%)	54 (66%)	
Live birth				0.742
No	77 (44%)	42 (45%)	35 (43%)	
Yes	98 (56%)	51 (55%)	47 (57%)	

### Univariate and multivariate logistic regression analyses

Univariate and multivariate logistic regression analyses were used to obtain the ORs and 95% CIs of the independent risk factors contributing to clinical pregnancy. The results revealed that a high AFC, basal FSH level, and AMH level and an endometrial thickness ≥8 mm were independent predictors of clinical pregnancy after FET. Protective factors for clinical pregnancy after FET cycles include a greater AFC (aOR, 1.04; 95% CI, 1.02–1.06; P<0.001) and an endometrial thickness ≥8 mm on the day of ovulation (aOR, 2.31; 95% CI, 1.09–4.93; P=0.029); risk factors for clinical pregnancy after FET cycles include a greater basal FSH level (aOR, 0.90; 95% CI, 0.81–0.99; P=0.038) and a greater AMH level (aOR, 0.94; 95% CI, 0.88–1.00; P=0.044); and other endometrial and subendometrial ultrasound indicators on the day of ovulation and the presence of uterine adhesions do not affect the clinical pregnancy rate (**Table 3**).

**Table 3:** Univariate and multivariate logistic regression analyses of risk factors contributing to clinical pregnancy.

Characteristic	Univariate Logistic Regression					Multivariate Logistic Regression			
	N	Event N	OR	95% CI	p value	Event N	OR	95% CI	p value
Age (years)	584	413	0.99	0.93, 1.05	0.710	388	1.04	0.97, 1.12	0.220
Duration of infertility (years)	572	402	0.96	0.89, 1.04	0.330	388	0.92	0.84, 1.00	0.053
BMI	584	413	1.07	0.96, 1.19	0.240	388	1.06	0.95, 1.19	0.320
Cycles	585	414	0.63	0.41, 0.98	0.040	388	0.69	0.42, 1.15	0.150
AFC	585	414	1.03	1.01, 1.04	<0.001	388	1.04	1.02, 1.06	<0.001
FSH	574	407	0.88	0.80, 0.95	0.004	388	0.90	0.81, 0.99	0.038
LH	575	407	0.99	0.97, 1.01	0.260	388	1.00	0.98, 1.02	0.730
AMH	572	405	1.01	0.96, 1.06	0.700	388	0.94	0.88, 1.00	0.044
Endometrial morphology classification	592	414				388			
Type A			—	—			—	—	
Type B+Type C			1.63	0.96, 2.73	0.067		1.27	0.66, 2.42	0.460
Endometrial blood flow classification	592	414				388			
I			—	—			—	—	
II			1.28	0.72, 2.24	0.390		1.03	0.51, 2.02	0.940
III			1.32	0.61, 2.84	0.480		0.98	0.37, 2.58	0.970
Endometrial thickness (mm)	592	414				388			
<8			—	—			—	—	
≥8			2.78	1.51, 5.13	<0.001		2.31	1.09, 4.93	0.029
Endometrial volume (ml)	592	414				388			
<2			—	—			—	—	
≥2			2.10	1.30, 3.37	0.002		1.25	0.62, 2.51	0.530
Frequency of endometrial peristalsis (times/min)	591	413				388			
<2			—	—			—	—	
≥2			1.56	1.05, 2.30	0.026		1.08	0.66, 1.76	0.760
VI	588	410	1.00	0.94, 1.07	>0.990	388	0.97	0.87, 1.08	0.540
FI	588	410	0.99	0.96, 1.02	0.530	388	0.99	0.95, 1.03	0.730
VFI	588	410	1.01	0.92, 1.13	0.920	388	0.99	0.86, 1.21	0.900
Subendometrial volume (ml)	592	414	1.08	1.01, 1.15	0.019	388	1.02	0.93, 1.11	0.700
Subendometrial VI	588	410	1.02	0.99, 1.05	0.200	388	1.01	0.88, 1.09	0.790
Subendometrial FI	588	410	1.00	0.96, 1.04	0.870	388	0.97	0.91, 1.02	0.240
Subendometrial VFI	588	410	1.06	0.99, 1.15	0.110	388	1.10	0.95, 1.64	0.380
group	592	414				388			
Non-IUAs			—	—			—	—	
IUAs			0.70	0.48, 1.03	0.066		0.89	0.55, 1.44	0.630

OR, odds Ratio; CI, confidence Interval; BMI, body mass index; AFC, antral follicle count; FSH, follicle-stimulating hormone; LH, luteinizing hormone; AMH, anti-Müllerian hormone; VI, vascularization index; FI, flow index; VFI, vascularization-flow index; IUA, intrauterine adhesion.

## Discussion

In this study, we compared ER on the day of ovulation between patients with and without a history of IUAs and reported that the live birth rate was significantly higher in patients without IUAs than in patients with IUAs. Compared with patients without IUAs, patients with IUAs (regardless of pregnancy status) had a thinner endometrium, a lower likelihood of having Type B+C endometrial morphology, a smaller volume, a lower frequency of endometrial peristalsis, a smaller subendometrial volume, and less subendometrial blood perfusion. However, there was no significant difference in the live birth rate between patients with mild and moderate–severe adhesions. Compared with patients with moderate–severe adhesions, those with mild adhesions had greater endometrial thickness and volume, peristaltic frequency, subendometrial volume and subendometrial blood perfusion. A high AFC and an endometrial thickness ≥8 mm are protective factors for clinical pregnancy, while high levels of FSH and AMH are risk factors for clinical pregnancy.

IUA development is multifactorial with multiple predisposing and causal factors, which often cause subfertility or infertility (21). Currently, hysteroscopic adhesiolysis is widely considered the gold standard for the diagnosis and treatment of IUAs (22). TVS is a first-line diagnostic method for IUAs and a noninvasive modality for analyzing ER (23).

In this study, IUAs affected the endometrial thickness, volume, morphology, subendometrial blood perfusion and endometrial peristalsis. Some previous studies have suggested that the “triple line sign” on the day of ovulation is related to clinical pregnancy and that adhesion disrupts the morphology of the endometrium (24, 25); thus, the proportion of patients with the triple line sign also decreases significantly (Types B+C). The live birth rate of patients with IUAs was lower, which was consistent with the results of previous studies (26, 27), but there was no significant difference in the clinical pregnancy rates, indicating that IUAs may have little effect on embryo implantation but may impair the ability to tolerate pregnancy in the long term.

Notably, there was no significant difference in the ultrasound parameters of patients without IUAs, regardless of pregnancy status. Among patients with IUAs, pregnant patients had significantly better endometrial ultrasound indicators (thickness, volume, blood perfusion) than nonpregnant patients did. However, there was no significant difference in peristaltic waves between pregnant and nonpregnant patients with adhesions, which is likely due to adhesion affecting the flexibility of endometrial peristalsis. However, we observed that there was no significant difference in endometrial blood perfusion between patients with adhesions and those without adhesions. This may be because, in this study, relatively few patients had moderate–severe adhesions, and most of these patients had moderate adhesions; thus, there was no significant difference in blood perfusion between patients with mild and moderate–severe adhesions.

Studies generally suggest that moderate to severe IUAs have a greater impact on pregnancy outcomes (27, 28). Although the ER indicators of moderate–severe adhesions were better than those of mild adhesions in this study, there was no significant difference in the live birth rate or clinical pregnancy rate between these two groups. This may be related to the high proportion of moderate adhesions, or it may indicate that patients with adhesions can achieve satisfactory pregnancy outcomes with proper treatment before transfer.

When ovarian reserve function and endometrial development are good, the probability of clinical pregnancy is relatively high. Similarly, decreased ovarian function (elevated FSH) is a risk factor for clinical pregnancy, which is consistent with previous research results (29, 30). Notably, the results of this study indicate that a high AMH level is a risk factor for clinical pregnancy.

The present work has several significant strengths. First, this was the first prospective study comparing ER on the day of ovulation during natural cycles between patients with and without a history of IUAs. Second, the degree of influence of mild and moderate–severe adhesions was also analyzed. However, several limitations remain. First, this study analyzed only the ER status of the endometrium on the day of ovulation in natural FET cycles, and the impact of adhesions on other populations and nonovulation days remains to be verified. Second, among patients with moderate-severe IUAs, the proportion of those with severe adhesions is relatively small, which may have a certain impact on the observation results. Third, no comparison of ER was made preadhesiolysis and postadhesiolysis.

In conclusion, IUAs can affect ER on the day of ovulation and the live birth rate during natural cycles. Moderate–severe IUAs have a greater impact on ER than mild IUAs do; however, if these adhesions are treated properly, they do not have adverse effects on the clinical pregnancy rate. A high AFC and an endometrial thickness ≥8 mm are protective factors for clinical pregnancy, whereas high FSH and AMH levels are risk factors for clinical pregnancy. However, the conclusions and ideas of such reviews need to be validated in other populations, as well as in studies with larger sample sizes and longer follow-up periods.

## Data Availability

The original contributions presented in the study are included in the article/supplementary material. Further inquiries can be directed to the corresponding author.
