# Corrigendum: The impact of intrauterine adhesions on endometrial receptivity in patients undergoing *in vitro* fertilization-embryo transfer

**DOI:** 10.3389/fendo.2025.1585477

**Published:** 2025-03-27

**Authors:** Yan Ouyang, Yangqin Peng, Mingxiang Zheng, Yuyao Mao, Fei Gong, Yuan Li, Hui Chen, Xihong Li

**Affiliations:** ^1^ Clinical Research Center for Reproduction and Genetics in Hunan Province, Reproductive & Genetic Hospital of CITIC-Xiangya, Changsha, China; ^2^ College of Information Science and Engineering, Hunan University, Changsha, China; ^3^ NHC Key Laboratory of Human Stem Cell and Reproductive Engineering, School of Basic Medical Sciences, Central South University, Hunan University, Changsha, China

**Keywords:** endometrial receptivity, intrauterine adhesion, frozen-thawed embryo transfer, natural cycle, ovulation day, transvaginal sonography

In the published article, there were errors in **Tables 1**, **2** as published. The corrected **Tables 1**, **2** and their captions “Table 1 Comparisons of characteristics and ultrasound parameters on the day of ovulation/Table 2 Comparisons of characteristics and ultrasound parameters on the day of ovulation between the mild- and moderate-severe IUA groups” appear below.

**Table 1:** Comparisons of characteristics and ultrasound parameters on the day of ovulation.

Variable	Non-IUAs			IUAs			p value				
	1	2	3	4	5	6	1 vs. 4	2 vs. 3	5 vs. 6	2 vs. 5	3 vs. 6
	Overall	Nonpregnant group	Pregnant group	Overall	Nonpregnant group	Pregnant group					
Cycles	417	116	301	175	62	113					
Baseline characteristics											
Age (years)	30.22 ± 3.17	30.19 ± 3.34	30.23 ± 3.12	30.34 ± 2.84	30.56 ± 2.72	30.22 ± 2.90	0.911	0.814	0.447	0.641	0.786
Duration of infertility (years)	3.59 ± 2.35	3.93 ± 2.91	3.47 ± 2.11	2.91 ± 1.88	2.84 ± 1.88	2.94 ± 1.89	<0.001	0.368	0.693	0.012	0.017
BMI (kg/m2)	21.20 ± 1.71	21.00 ± 1.74	21.28 ± 1.69	20.94 ± 1.67	20.99 ± 1.49	20.91 ± 1.77	0.067	0.082	0.605	0.903	0.034
Number of oocyte retrieval cycles	1.14 ± 0.37	1.21 ± 0.43	1.11 ± 0.35	1.17 ± 0.42	1.18 ± 0.43	1.16 ± 0.41	0.555	0.011	0.737	0.535	0.301
AFC	18.18 ± 14.57	15.03 ± 11.85	19.32 ± 15.29	19.57 ± 14.38	15.60 ± 17.07	21.74 ± 12.21	0.190	0.024	<0.001	0.476	0.022
FSH	5.62 ± 2.31	6.19 ± 2.94	5.42 ± 2.01	5.88 ± 2.03	6.08 ± 2.00	5.77 ± 2.05	0.098	0.001	0.327	0.935	0.075
LH	5.34 ± 10.96	6.25 ± 11.54	5.01 ± 10.75	5.05 ± 6.02	5.53 ± 7.26	4.79 ± 5.24	0.014	0.107	0.782	0.401	0.023
AMH	4.92 ± 3.78	4.88 ± 3.39	4.94 ± 3.92	5.14 ± 3.63	4.91 ± 3.97	5.26 ± 3.44	0.354	0.701	0.152	0.534	0.102
Ultrasound indicators on the day of ovulation											
Endometrial morphology classification							<0.001	0.66	0.034	<0.001	<0.001
Type A	25 (6.0%)	6 (5.2%)	19 (6.3%)	43 (25%)	21 (34%)	22 (19%)					
Type B+Type C	392 (94%)	110 (95%)	282 (94%)	132 (75%)	41 (66%)	91 (81%)					
Endometrial blood flow classification							0.070	0.780	0.197	0.026	0.702
I	37 (8.9%)	10 (8.6%)	27 (9.0%)	23 (13%)	11 (18%)	12 (11%)					
II	330 (79%)	90 (78%)	240 (80%)	140 (80%)	49 (79%)	91 (81%)					
III	50 (12%)	16 (14%)	34 (11%)	12 (6.9%)	2 (3.2%)	10 (8.8%)					
Endometrial thickness (mm)	11.21 ± 2.08	11.29 ± 2.23	11.18 ± 2.02	9.53 ± 1.93	9.57 ± 2.41	9.50 ± 1.62	<0.001	0.613	0.767	<0.001	<0.001
<8	12 (2.9%)	4 (3.4%)	8 (2.7%)	34 (19%)	20 (32%)	14 (12%)					
≥8	405 (97%)	112 (97%)	293 (97%)	141 (81%)	42 (68%)	99 (88%)	<0.001	0.745	0.001	<0.001	<0.001
Endometrial volume (ml)	4.21 ± 1.72	4.11 ± 1.81	4.24 ± 1.69	2.89 ± 1.45	2.63 ± 1.41	3.03 ± 1.46	<0.001	0.415	0.049	<0.001	<0.001
<2	26 (6.2%)	9 (7.8%)	17 (5.6%)	57 (33%)	28 (45%)	29 (26%)					
≥2	391 (94%)	107 (92%)	284 (94%)	118 (67%)	34 (55%)	84 (74%)	<0.001	0.424	0.008	<0.001	<0.001
Frequency of endometrial peristalsis (time/min)	2.85 ± 1.39	2.87 ± 1.48	2.84 ± 1.36	1.79 ± 1.42	1.57 ± 1.34	1.91 ± 1.46	<0.001	0.767	0.127	<0.001	<0.001
<2	67 (16%)	21 (18%)	46 (15%)	83 (47%)	35 (56%)	48 (42%)					
≥2	349 (84%)	95 (82%)	254 (85%)	92 (53%)	27 (44%)	65 (58%)	<0.001	0.491	0.077	<0.001	<0.001
Endometrial VI	2.13 ± 2.96	2.19 ± 2.67	2.10 ± 3.06	1.95 ± 2.79	1.85 ± 2.28	2.00 ± 3.05	0.228	0.458	0.349	0.126	0.696
Endometrial FI	28.04 ± 6.71	28.86 ± 6.33	27.72 ± 6.83	28.83 ± 6.10	27.92 ± 6.63	29.33 ± 5.75	0.509	0.426	0.041	0.224	0.078
Endometrial VFI	0.81 ± 2.10	0.79 ± 1.19	0.81 ± 2.37	0.69 ± 1.19	0.69 ± 1.31	0.68 ± 1.12	0.460	0.296	0.251	0.122	0.855
Subendometrial volume (ml)	11.54 ± 2.94	11.30 ± 2.82	11.62 ± 2.99	9.57 ± 2.35	9.06 ± 2.36	9.86 ± 2.30	<0.001	0.205	0.046	<0.001	<0.001
Subendometrial VI	7.47 ± 6.60	7.47 ± 5.79	7.48 ± 6.90	6.74 ± 6.46	5.32 ± 5.22	7.52 ± 6.95	0.056	0.55	0.031	0.005	0.759
Subendometrial FI	32.36 ± 4.76	32.84 ± 5.10	32.17 ± 4.61	32.39 ± 4.01	31.62 ± 4.17	32.81 ± 3.87	0.937	0.282	0.053	0.103	0.176
Subendometrial VFI	2.70 ± 3.10	2.57 ± 2.06	2.75 ± 3.42	2.23 ± 2.23	1.71 ± 1.72	2.52 ± 2.42	0.033	0.483	0.023	0.002	0.688
Clinical pregnancy							0.065				
No	116 (28%)			62 (35%)							
Yes	301 (72%)			113 (65%)							
Live birth							0.039				
No	146 (35%)			77 (44%)							
Yes	271 (65%)			98 (56%)							

**Table 2:** Comparisons of characteristics and ultrasound parameters on the day of ovulation between the mild- and moderate-severe IUA groups.

Variable	Overall	Moderate-to-severe IUAs	Mild IUAs	p value
Cycles	175	93	82	
Baseline characteristics				
Age (years)	30.34 ± 2.84	30.24 ± 2.89	30.47 ± 2.79	0.743
Duration of infertility (years)	2.91 ± 1.88	2.81 ± 1.72	3.01 ± 2.05	0.739
BMI (kg/m2)	20.94 ± 1.67	20.72 ± 1.52	21.20 ± 1.80	0.07
Number of oocyte retrieval cycles	1.17 ± 0.42	1.18 ± 0.44	1.15 ± 0.39	0.606
AFC	19.57 ± 14.38	17.62 ± 13.86	21.77 ± 14.73	0.057
FSH	5.88 ± 2.03	5.80 ± 2.13	5.98 ± 1.93	0.266
LH	5.05 ± 6.02	5.40 ± 7.42	4.66 ± 3.87	0.518
AMH	5.14 ± 3.63	4.82 ± 3.16	5.49 ± 4.08	0.446
Ultrasound indicators on the day of ovulation				
Endometrial morphology classification				0.958
Type A	43 (25%)	23 (25%)	20 (24%)	
Type B+Type C	132 (75%)	70 (75%)	62 (76%)	
Endometrial blood flow classification				0.864
I	23 (13%)	13 (14%)	10 (12%)	
II	140 (80%)	73 (78%)	67 (82%)	
III	12 (6.9%)	7 (7.5%)	5 (6.1%)	
Endometrial thickness (mm)	9.53 ± 1.93	9.07 ± 1.69	10.05 ± 2.07	<0.001
<8	34 (19%)	25 (27%)	9 (11%)	0.008
≥8	141 (81%)	68 (73%)	73 (89%)	
Endometrial volume (ml)	2.89 ± 1.45	2.59 ± 1.29	3.23 ± 1.56	0.002
<2	57 (33%)	39 (42%)	18 (22%)	0.005
≥2	118 (67%)	54 (58%)	64 (78%)	
Frequency of endometrial peristalsis (time/min)	1.79 ± 1.42	1.43 ± 1.26	2.20 ± 1.49	<0.001
<2	83 (47%)	54 (58%)	29 (35%)	0.003
≥2	92 (53%)	39 (42%)	53 (65%)	
Endometrial VI	1.95 ± 2.79	2.13 ± 3.14	1.73 ± 2.34	0.311
Endometrial FI	28.83 ± 6.10	28.15 ± 6.37	29.60 ± 5.72	0.367
Endometrial VFI	0.69 ± 1.19	0.72 ± 1.29	0.65 ± 1.06	0.537
Subendometrial volume (ml)	9.57 ± 2.35	9.22 ± 2.29	9.97 ± 2.36	0.023
Subendometrial VI	6.74 ± 6.46	6.69 ± 6.19	6.79 ± 6.79	0.98
Subendometrial FI	32.39 ± 4.01	31.48 ± 3.64	33.43 ± 4.17	0.002
Subendometrial VFI	2.23 ± 2.23	2.14 ± 2.02	2.33 ± 2.45	0.842
Clinical pregnancy				0.739
No	62 (35%)	34 (37%)	28 (34%)	
Yes	113 (65%)	59 (63%)	54 (66%)	
Live birth				0.742
No	77 (44%)	42 (45%)	35 (43%)	
Yes	98 (56%)	51 (55%)	47 (57%)	

The authors apologize for this error and state that this does not change the scientific conclusions of the article in any way. The original article has been updated.

